# Immense variability in the sea surface temperature near macro tidal flat revealed by high-resolution satellite data (Landsat 8)

**DOI:** 10.1038/s41598-021-04465-4

**Published:** 2022-01-07

**Authors:** Seung-Tae Lee, Yang-Ki Cho, Duk-jin Kim

**Affiliations:** School of Earth and Environmental Sciences, Seoul National University, Seoul, Korea

**Keywords:** Environmental impact, Physical oceanography

## Abstract

Sea surface temperature (SST) is crucial for understanding the physical characteristics and ecosystems of coastal seas. SST varies near the tidal flat, where exposure and flood recur according to the tidal cycle. However, the variability of SST near the tidal flat is poorly understood owing to difficulties in making in-situ observations. The high resolution of Landsat 8 enabled us to determine the variability of SST near the macro tidal flat. The spatial distribution of the SST extracted from Landsat 8 changed drastically. The seasonal SST range was higher near the tidal flat than in the open sea. The maximum seasonal range of coastal SST exceeded 23 °C, whereas the range in the open ocean was approximately 18 °C. The minimum and maximum horizontal SST gradients near the tidal flat were approximately − 0.76 °C/10 km in December and 1.31 °C/10 km in June, respectively. The heating of sea water by tidal flats in spring and summer, and cooling in the fall and winter might result in a large horizontal SST gradient. The estimated heat flux from the tidal flat to the seawater based on the SST distribution shows seasonal change ranging from − 4.85 to 6.72 W/m^2^.

## Introduction

The sea surface temperature (SST) field provides information on the surface current and water mass^1,2^. SST plays an important role in the exchange of energy, momentum, and moisture between the ocean and the atmosphere^3,4^. SST substantially affects the dynamic process and ecosystems in the coastal region^5^.

SST in coastal regions with macro tidal flats may be greatly affected by the heat exchange between the tidal flat and seawater^6–8^. The tidal flat is located between the coastlines during high tide and low tide, experiences repeated exposure to atmosphere and flooding according to the tidal phase^9^. Tidal flats are unique environments for various populations, such as migratory birds, crabs, and mollusks^10,11^. These biomes are subjected to complicated changes in temperature via heat exchange, not only between air and seawater but also between the sediment and seawater^12,13^.

Some studies have been conducted to elucidate the complicated changes in water temperature in tidal flat regions. The daily heat content of the sediment in the tidal flat on the western coast of the Dutch Wadden Sea changed as the tidal cycle changed, resulting in a 15-days periodicity in seawater temperature^6^. The effect of tidal flats on seawater has been studied using a numerical model on the west coast of Korea^14^. The water temperature in the tidal flat region has semidiurnal variations on the southwest coast of Korea^7^. The amount of heat exchange was estimated based on the tidal phase in the tidal flat region using a three-dimensional numerical model^8^.

Despite previous studies on the change in water temperature in the tidal flat region, the spatiotemporal variation in this region is poorly understood owing to the difficulty in access. The tidal flat is too shallow to measure by vessel. We addressed this limitation using satellite-observed data. There have been a few previous studies to investigate the SST near coastal regions using satellite data. They mainly used the National Oceanic and Atmospheric Administration (NOAA) Advanced Very High Resolution Radiometer (AVHRR) measurements to study the coastal phenomena^15–18^. They did not, however, investigate the narrow SST structure in the tidal flat region because the spatial resolution of AVHRR was more than over 1.1 km. Landsat satellite data with high resolution provide detailed information on topography and SST in the tidal flat region. Landsat is a program jointly developed by the United States Geological Survey (USGS) and the National Aeronautics and Space Administration (NASA) to continuously observe the Earth using satellites^19^. It is a polar orbit satellite and obtains high-resolution images (30 m resolution of visible and near infrared band, and 60 m or 100 m resolution of thermal infrared band). The Landsat data have been used to extract the waterline of Gomso Bay located on the west coast of Korea^16^ and coast of China^20^.

Landsat satellite data enable us to distinguish among sea, tidal flat, landmass, and coastal lines and to estimate surface temperatures of sea and land from brightness temperature. This can allow a high-resolution SST distribution in coastal seas. SST variability and SST gradient were quantified by using Landsat TM band 6 thermal infrared images on the central coast of Maine^2^. Climate observations of SST from Landsat TM and ETM + thermal infrared data showed that isolated and shallow waters had larger temperature variations than well-connected embayments or coastal oceans^5^.

**Figure 1:**
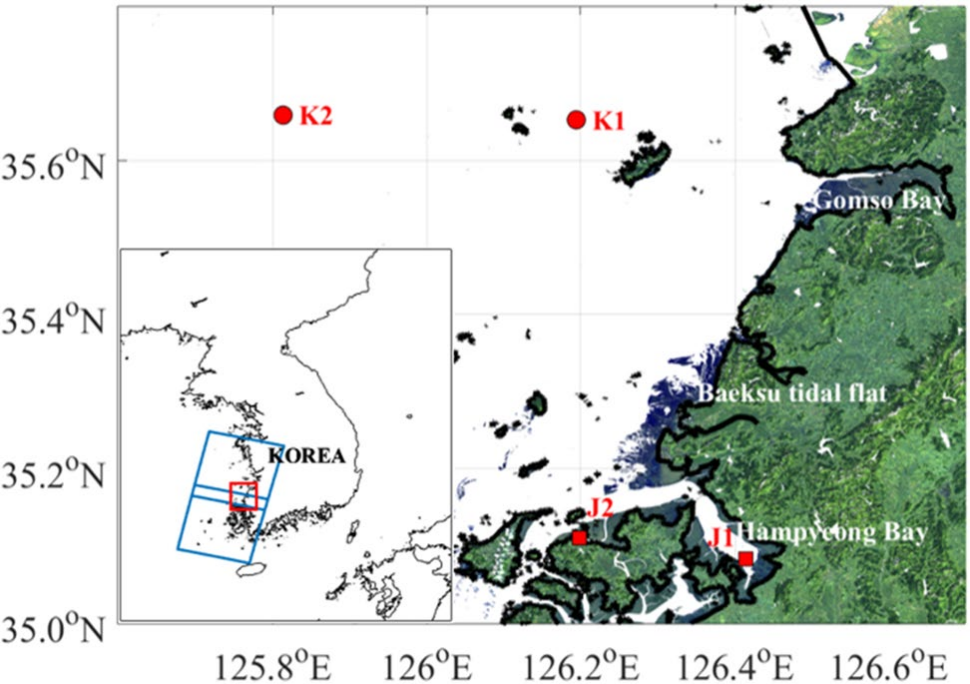
True color composite (RGB) image from Landsat 8 on February 21, 2019 in the study area. Blue boxes represent the boundary of Landsat 8 scene and the red box represents the study area. Red circles (K1 and K2) and red squares (J1 and J2) represent the location of buoy operated by Korea Hydrographic and Oceanographic Agency (KHOA) and JNSI, respectively. Black line and gray area represent the coastal line and tidal flat, respectively. Figures were generated by S.-T. Lee using MATLAB R2020a (http://www.mathworks.com).

Although many studies based on satellite-observed data have reported the spatial variations of SST in the coastal sea, the variability of the water temperature near a macro tidal flat is ill-understood. In this study, the characteristics of the SST distribution on the west coast of Korea were analyzed based on Landsat 8 data (Fig. 1). The west coast of Korea is one of the regions where the tidal flat is widely distributed owing to the area’s large tidal range and shallow water depth. Data from Landsat 8 from 2013 were analyzed to calculate SST using the split-window algorithm for bands 10 and 11.

## Results and discussion

### Large seasonal variability of SST near tidal flat

**Figure 2:**
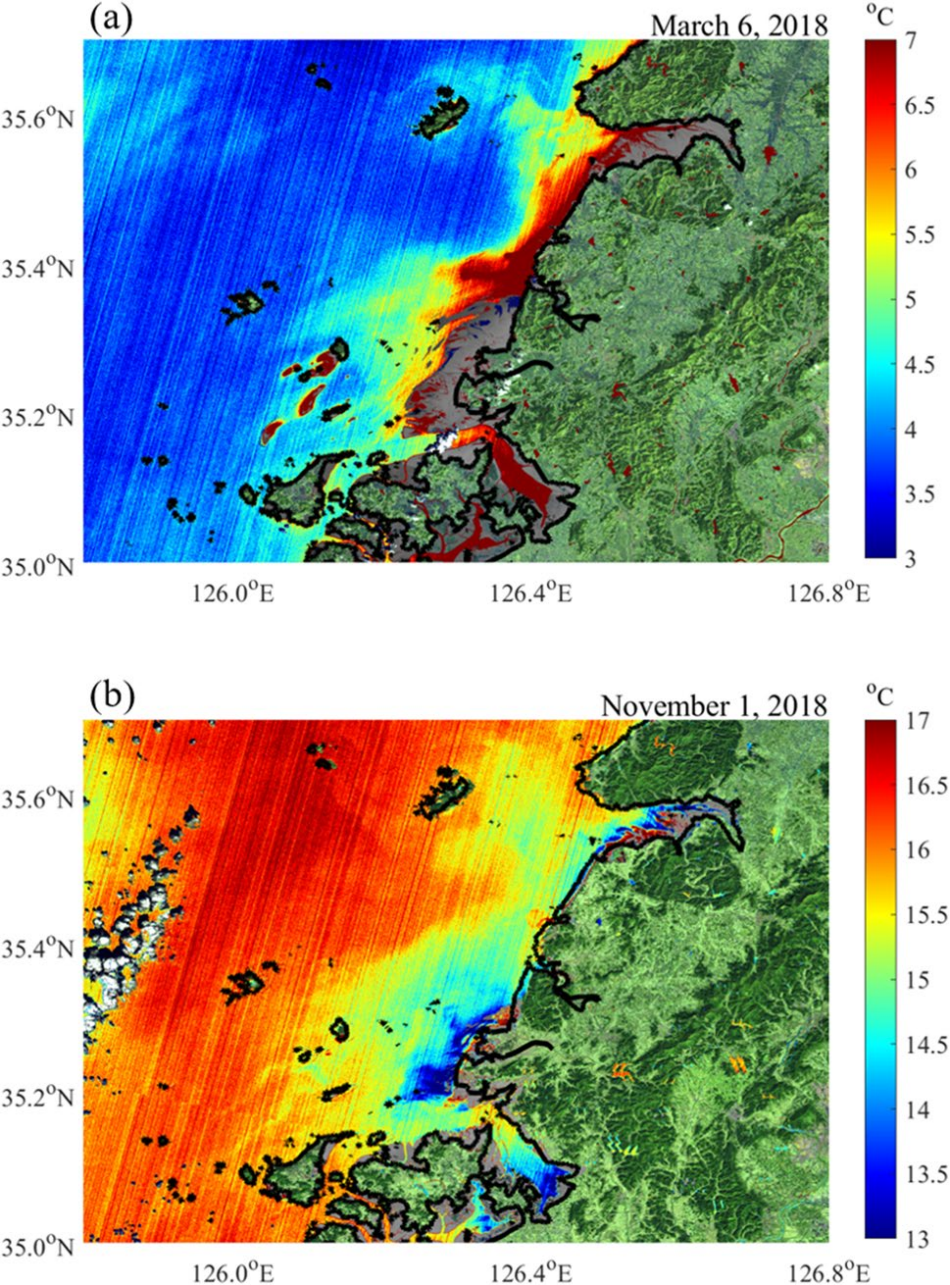
Sea surface temperature derived from Landsat 8 on (**a**) March 6, 2018 and (**b**) November 1, 2018. The pixels corresponding to the tidal flat are marked in gray. The pixels corresponding to the land are marked as RGB of the Landsat 8. The thick solid line represents the coastline. The clouds are presented in white. Figures were generated by S.-T. Lee using MATLAB R2020a (http://www.mathworks.com).

SST from Landsat 8 was analyzed to examine the seasonal variation in the SST near the macro tidal flat. Figure 2 shows the horizontal distribution of SST on March 6, 2018, and November 1, 2018, which represent the heating and cooling seasons, respectively. Most of the tidal flats were exposed in each scene. SSTs near the Baeksu tidal flat, Hampyeong Bay, and Gomso Bay were warmer than those in the open sea in March but colder in November.

**Figure 3:**
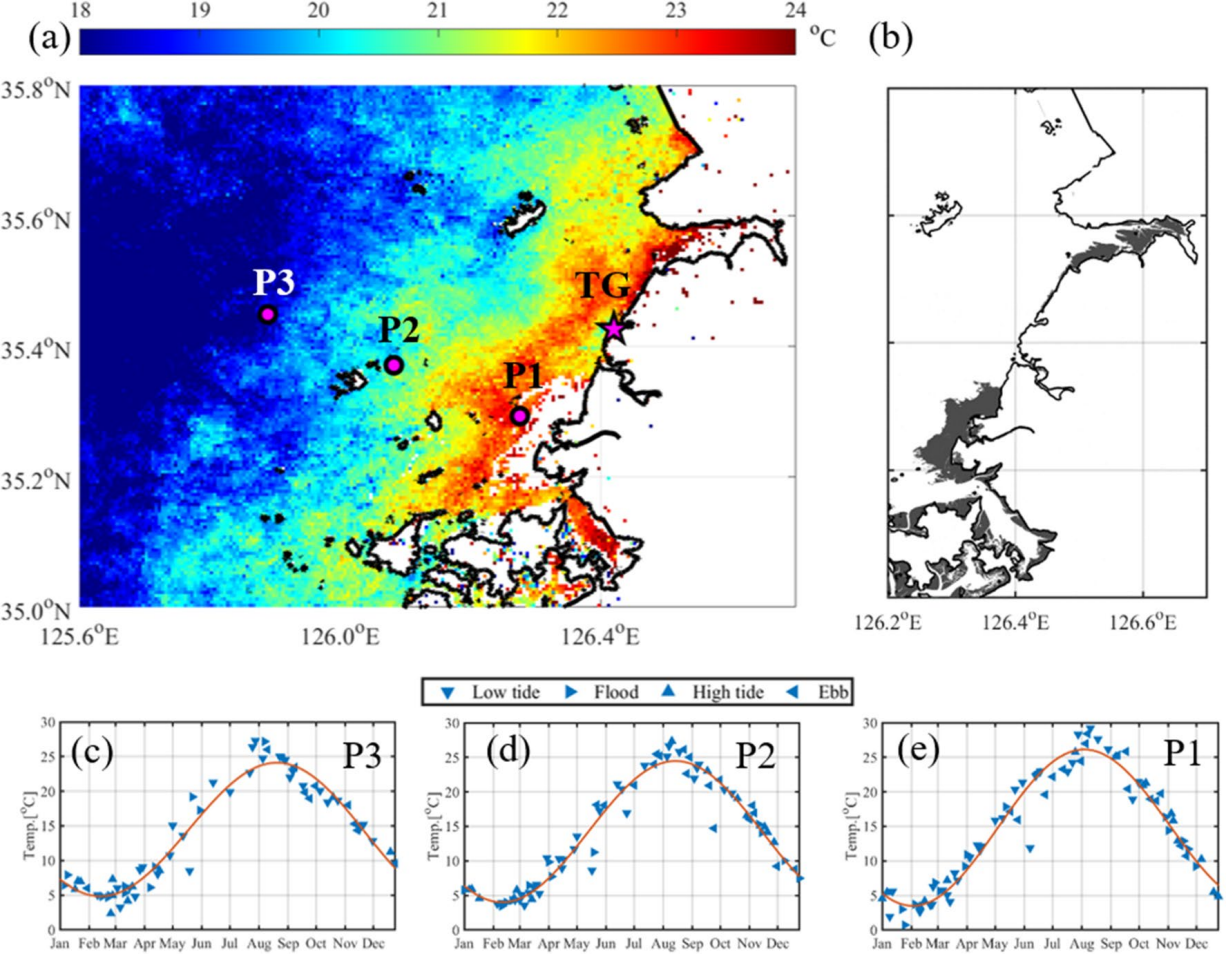
Horizontal seasonal range of the sea surface temperature near the tidal flat and a time series of sea surface temperature at different points. (**a**) Seasonal range of the sea surface temperature calculated from the difference between the maximum and minimum temperatures of the fitted seasonal variation at each pixel. The magenta star represents the Yeonggwang tidal station. (**b**) Distribution of the tidal flat in the study area. Tidal flats are marked in gray. Time series of sea surface temperature at grid points (**c**) P3, (**d**) P2, and (**e**) P1. Blue triangles represent the tidal period when the SST was retrieved. Downward, rightward, upward, and leftward triangles represent the low tide, flood, high tide and Ebb period, respectively. The red line represents a fitted SST curve with a 12th order polynomial equation. Figures were generated by S.-T. Lee using MATLAB R2020a (http://www.mathworks.com).

The retrieved SST from Landsat 8 was interpolated at each grid with a resolution of 0.005°. All available data at each grid were arranged on the Julian day to investigate seasonal variation, regardless of the observed year. Three continuous years of repeated data were fitted into a 12th-order polynomial. The fitted data in the central year were selected for analysis^5^. The tidal flat and land were excluded from the calculation. The seasonal SST range was calculated from the difference between the maximum and minimum temperatures from the fitted curve for each grid. The seasonal range of SST from Landsat 8 was calculated (Fig. 3). A large range of SST was observed near the tidal flat in the seasonal variation (Fig. 3a). The wider the tidal flat distribution, the larger the seasonal range (Fig. 3b). The seasonal range of SST near the tidal flat was larger by 5 °C than that in the open sea. The minimum range in the open ocean is about 18 °C. However, the maximum range of coastal SST exceeds 23 °C, which is significantly greater than the 14 °C in the bay of Southern New England^5^.

Three grid points: P1 (126.28° E, 35.29° N), P2 (126.08° E, 35.37° N), and P3 (125.89° E, 35.45° N), were selected for the spatial comparison of the annual variations in SST (Fig. 3c,d). The temperature ranges were 22.6 °C, 20.46 °C, and 19.27 °C at grid points P1, P2, and P3, respectively. The maximum temperature at P1 was the highest among the three grid points, but the minimum temperature was the lowest. The maximum and minimum temperatures at P1 appeared 17 days earlier compared to P3. The closer to the tidal flat, the higher the maximum SST and the lower the minimum SST, resulting in an increase in seasonal range. The maximum and minimum temperatures near the tidal flat appeared earlier than they do in the open sea.

### Cause of large variability in SST near the tidal flat

**Figure 4:**
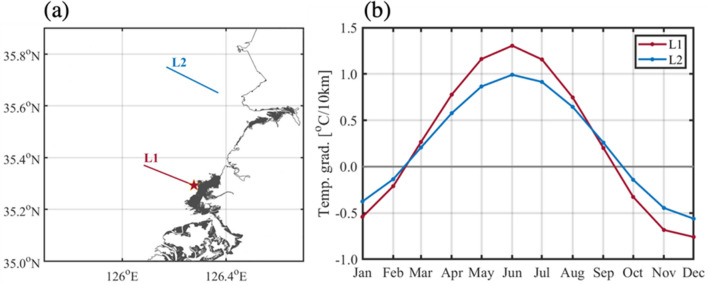
(**a**) Lines selected for the calculation of horizontal temperature gradient. Tidal flats are marked as gray. (**b**) Horizontal temperature gradient per 10 km derived from Landsat 8 along line L1 (red) near the tidal flat and along line L2 (blue) in far proximity from the tidal flat. Figures were generated by S.-T. Lee using MATLAB R2020a (http://www.mathworks.com).

A large horizontal SST gradient attributed to spatially uneven heating or cooling in the coastal seas has been reported in previous studies^2,5,21–23^. The large variability in the SST near the macro tidal flat might have affected the horizontal SST gradient in our study area. Two lines were selected to estimate the effect of the tidal flat on the SST variability: line L1 near the Baeksu tidal flat and line L2 far from the tidal flat (Fig. 4a). The horizontal SST gradient along each line was calculated for each month (Fig. 4b). The red and blue lines represent the horizontal SST gradients along lines L1 and L2, respectively. A negative value means that SST decreases onshore, and a positive value that SST increases onshore. The SST in both lines commonly decreased onshore in fall and winter (January, February, October, November, and December), but increased in spring and summer. However, the seasonal variation of the SST gradient along line L1 was remarkably larger than that along line L2. In winter, the coastal water temperature in line L1 was slightly lower than that in line L2, but higher in summer. The minimum and maximum gradients along line L1 were approximately − 0.76 °C/10 km in December and approximately 1.31 °C/10 km in June, respectively. These gradients are significantly larger than the minimum (− 0.44 °C/10 km) in January and the maximum (0.5 °C/10 km) in July on the coast of Maine^2^. The minimum and maximum gradients along line L2 were approximately − 0.56 °C/10 km in December, and approximately 0.99 °C/10 km in June, respectively.

Certain studies on the heat exchange between tidal flats and seawater have been conducted in areas where tidal flats are widely distributed^6–8,14^. The seasonal difference between the water temperature gradients of lines L1 and L2 might be due to the heat exchange between the tidal flat and sea water. The water temperature was vertically homogeneous in the study area owing to active vertical mixing by strong tidal currents. The SST depends on the water depth in a well-mixed shallow sea where the advection effect is not significant^5,22,24^.

The horizontal temperature gradient in line L1 was remarkably larger despite a similar gradient of water depth. The calculated gradient of water depth along each line calculated using gridded bathymetric data of 30 s was 5.49 m/10 km for line L1, and 5.61 m/10 km for line L2^25^. The larger gradient along line L1 despite the similar water depth gradient implies that the tidal flat near line L1 acts as a sink or source of heat.

**Figure 5:**
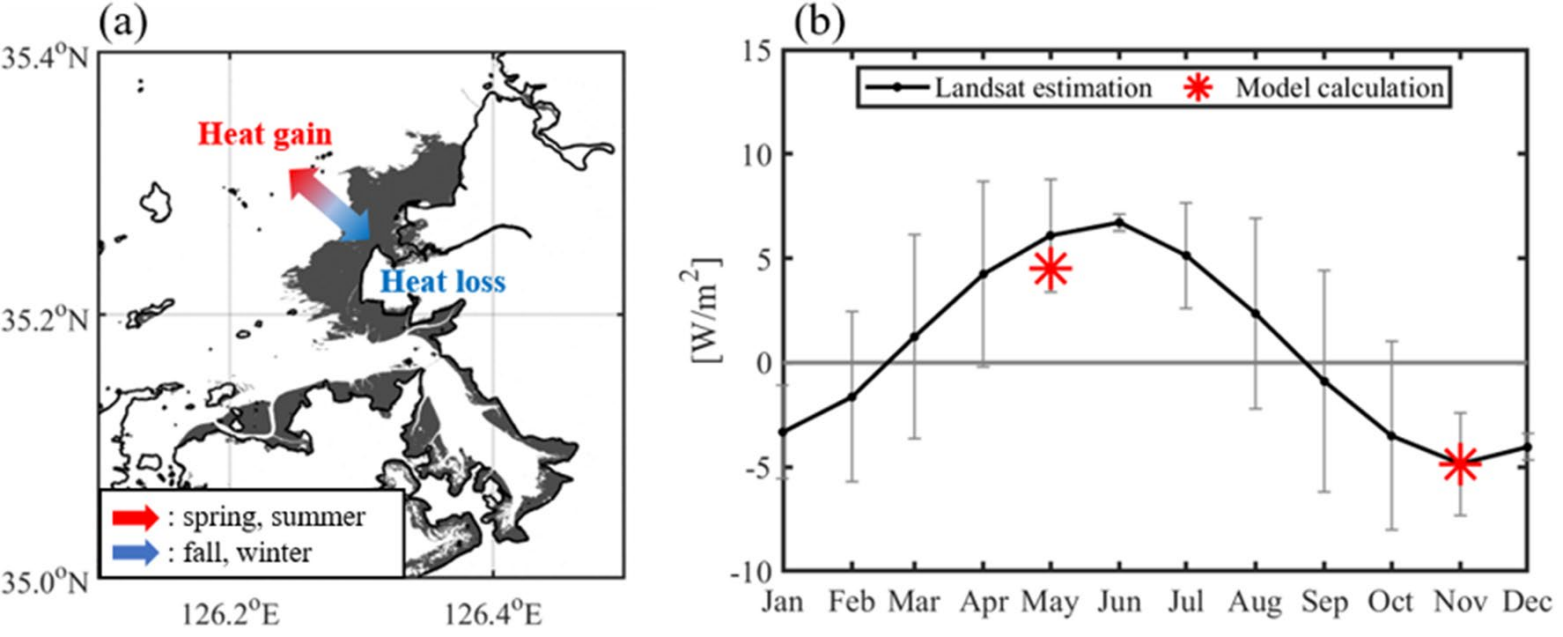
(**a**) Schematic diagram of heat exchange between the tidal flat and the seawater. Red arrow represents that sea water gain heat from tidal flat in spring and summer. Blue arrow represents that sea water loss heat to tidal flat in fall and winter. (**b**) Monthly heat exchange between the tidal flat and seawater at the red point of L1 (Fig. 4) estimated from Landsat 8 data. The vertical bar represents one standard deviation in each month. The red star is the heat exchange between the seawater and tidal flat calculated in previous study^8^. Figures were generated by S.-T. Lee using MATLAB R2020a (http://www.mathworks.com).

The additional heat at the onshore end point (red star in Fig. 4a) of line L1 was estimated from the horizontal SST gradient difference between the two lines. The additional heat flux, which causes a relatively large horizontal gradient of water temperature in line L1, was estimated based on the relative water temperature difference between two onshore end points in both lines. The estimated additional heat flux at the end point of line L1 for each month is shown in Fig. 5b. The black line represents the required monthly heat flux, and the gray vertical bars represent one standard deviation. In June, the standard deviation was low owing to the lack of usable scenes because of cloud contamination. The estimated heat flux shows a large seasonal variation (− 4.85 W/m^2^ to 6.72 W/m^2^). This implies that sea water gains heat from the tidal flat in spring and summer and loses heat to the tidal flat in fall and winter (Fig. 5a). A previous study^8^ calculated the heat exchange between Baeksu tidal flat and seawater using the unstructured grid, finite-volume coastal ocean model (FVCOM) with a code for calculating the sediment temperature according to the heat exchange between seawater and the seabed. Here, the tidal flat supplied maximum heat to the seawater in May, and the gained maximum heat from the seawater in November. The estimated heat fluxes are comparable with the calculated heat exchange between the tidal flat and seawater based on model calculation in May and November (red stars in Fig. 5b). The calculated heat exchange in our study area was 4.50 W m^−2^ in May and − 4.86 W m^−2^ in November^8^. The results reported by a previous model study^8^ were within one standard deviation of our estimation.

## Conclusions

Landsat 8 data enabled us to figure out the large variability of SST near the macro tidal flat on the west coast of Korea, which has not been accessed for in-situ observation. SSTs derived from three different methods were evaluated using in-situ data observed using buoys.

The seasonal range of SST near the tidal flat was approximately 23 °C, whereas it is approximately 18 °C in the open seas. The maximum and minimum water temperatures near the tidal flat appeared approximately one month earlier than they did in the open sea. The minimum and maximum gradients near the tidal flat were approximately − 0.76 °C/10 km in December and approximately 1.31 °C/10 km in June. However, those far from the tidal flat were approximately − 0.56 °C/10 km in December and approximately 0.99 °C/10 km in June, respectively. Seasonal changes in the horizontal SST gradient were high because of the heat exchange between the tidal flat and sea water. The estimated heat exchange between the tidal flat and seawater based on Landsat 8 data was comparable with that of a previous study based on model calculation. The estimated heat flux from the tidal flat to the seawater exhibited large seasonal variation, with a minimum of − 4.85 W/m^2^ in December and a maximum of 6.72 W/m^2^ in June.

Our study suggests that the extensive utilization of Landsat 8 in research in macro tidal flat areas is expected. However, additional efforts based on in-situ observations and numerical model experiments are required to support our findings.

## Methods

### Landsat 8 data processing

Data contaminated by clouds should be removed to obtain an accurate SST. Landsat 8 provides the band quality for each pixel. Pixel values of 2720, 2724, 2728, and 2732, regarded as cloud-free, were removed. However, cloud detection based on pixel quality is limited in the removal of sea fog and low clouds. RGB data were additionally examined to exclude pixels contaminated by clouds. After removing cloud pixels, the others were classified into land, tidal flat, and sea water. The brightness temperature of Landsat 8 was also compared with the buoy observation data to remove data contaminated by clouds.

Classifying Landsat data into seawater, tidal flats, and land for this study was crucial. The near infrared band is more beneficial to distinguish between water and other regions, because the reflectivity of water decreases while that of non-water increases as the visible band approaches the near infrared band. So, the regions with water and non-water could be easily distinguished through histograms of the near infrared band near a coastal area which shows a clear bimodal distribution. This is consistent with the results of a previous study investigating another tidal flat in South Korea^22^. Sometimes, in the case of a non-water region, the bimodal structure lacks clarity due to a lack of vegetation that causes the upland to also be included, rather than only the exposed tidal flat. To solve this problem, the upland area was removed by NDVI in advance. The Landsat normalized difference vegetation index (NDVI), useful for understanding vegetation density and changes in plant health, was used to define land (https://www.usgs.gov/). The NDVI is determined as follows:

1 $$NDVI=\frac{Band \,5-Band\, 4}{Band\, 5+Band\, 4}.$$

The digital number of the near infrared band of each Landsat 8 scene shows three peaks in a histogram. The three peaks correspond to the sea, tidal flat, and landmass in digital numbers. Pixels with digital numbers corresponding to the sea, tidal flat, and landmass were classified at each scene according to a previous method^26^. A previous study^26^ using the near infrared band reported that its accuracy was within about 69 m, which is sufficient to demarcate the tidal flat of this study.

Landsat 8, equipped with an Operational Land Imager (OLI) and a Thermal Infrared Sensor (TIRS), was launched on February 11, 2013. The TIRS of Landsat 8 comprises two thermal infrared channels and can correct atmospheric effects using a split-window algorithm^27^. Band 10 covers the wavelength range of 10.6–11.2 μm and band 11, 11.5–12.5 μm^28^. The split-window algorithm was used to calculate the land surface temperature (LST) or water surface temperature (WST) using the warm temperatures of the two bands^29–34^. In this study, SST using three different algorithms proposed by Rongali et al.^33^, Vanhellemenot^31^, and Jang and Park^32^, were compared with the observation data to determine the optimal coastal region temperature.

All Landsat 8 OLI/TIRS data were obtained from NASA and the United States Geological Survey (USGS) (https://earthexplorer.usgs.gov/). Two scenes (scene numbers 116,035 and 116,036), including the study area and (Fig. 1), from January 2014 to May 2021 were analyzed. The scenes were captured at approximately 11 am (LT) every 16 days. The brightness temperatures of bands 10 and 11 provided by the USGS were used in this study.

The algorithm proposed by Rongali et al.^33^ was adapted to obtain the SST as follows:

2 $${\varvec{S}}{\varvec{S}}{{\varvec{T}}}_{{\varvec{R}}}={{\varvec{B}}{\varvec{T}}}_{10}+{{\varvec{C}}}_{1}\left({\varvec{B}}{{\varvec{T}}}_{10}-{\varvec{B}}{{\varvec{T}}}_{11}\right)+{{\varvec{C}}}_{2}{\left({\varvec{B}}{{\varvec{T}}}_{10}-{\varvec{B}}{{\varvec{T}}}_{11}\right)}^{2}+{{\varvec{C}}}_{0}+\left({{\varvec{C}}}_{3}+{{\varvec{C}}}_{4}{\varvec{W}}\right)\left(1-{\varvec{m}}\boldsymbol{ }\right)+\left({{\varvec{C}}}_{5}+{{\varvec{C}}}_{6}{\varvec{W}}\right)\Delta {\varvec{m}}.$$

${{\varvec{B}}{\varvec{T}}}_{10}$ and ${{\varvec{B}}{\varvec{T}}}_{11}$ are the brightness temperatures (°C) of bands 10 and 11, respectively. ${{\varvec{C}}}_{0}$ to ${{\varvec{C}}}_{6}$ is the split-window (SW) coefficient value^33,35,36^. ${\varvec{m}}$ is the mean of the water surface emissivity (WSE) of the TIRS bands $({(\mathbf{W}\mathbf{S}\mathbf{E}}_{10}+{\varvec{W}}{\varvec{S}}{{\varvec{E}}}_{11})/2\boldsymbol{ })$, ${\varvec{W}}$ is the atmospheric water vapor content, and $\Delta {\varvec{m}}$ is the difference in the WSE (${\mathbf{W}\mathbf{S}\mathbf{E}}_{10}-{\varvec{W}}{\varvec{S}}{{\varvec{E}}}_{11})$. Regarding water vapor content, the split-window covariance-variance ratio method and 16 × 16 adjacent pixels were used for calculation at every scene and pixel^37^. The WSEs of bands 10 and 11 are 0.9926 and 0.9877, respectively^31^.

SST proposed by Vanhellemenot^31^ was calculated as follows:

3 $$\begin{aligned} SST_{V} = & b_{0} + \left( {b_{1} + b_{2} \frac{{1 - m}}{m} + b_{3} \frac{{\Delta m}}{{m^{2} }}} \right)\frac{{BT_{{10}} + BT_{{11}} }}{2} \\ & + \left( {b_{4} + b_{5} \frac{{1 - m}}{m} + b_{6} \frac{{\Delta m}}{{m^{2} }}} \right)\frac{{BT_{{10}} - BT_{{11}} }}{2} + b_{7} \left( {BT_{{10}} - BT_{{11}} } \right)^{2} \\ \end{aligned}$$

The ${\text{b}}_{\text{n}}$(n = 0 to 7) coefficients were derived from simulations by Du et al.^34^ based on the ranges of column water vapor ($\text{g}/{\text{cm}}^{2}$). In this study, we used coefficient ${\text{b}}_{\text{n}}$ ranging from 0 to 6.3 $\text{g}/{\text{cm}}^{2}$.

A formula for obtaining multi-channel SST (MCSST) from Landsat 8 data through a matchup with buoy data in the coastal sea of the Korean peninsula was proposed by Jang and Park^32^ from April 2013 to August 2017. Like the previous two algorithms, the MCSST algorithm can compute SSTs in two independent ways. MCSST1, which uses only the brightness temperatures of bands 10 and 11 among MCSSTs, was selected for this study. Following is the MCSST1 formula:

4 $$SS{T}_{J}={a}_{1}B{T}_{11}+{a}_{2}\left(B{T}_{10}-B{T}_{11}\right)+{a}_{3}.$$

${a}_{1}$, ${a}_{2}$, and ${a}_{3}$ are 0.9767, 1.8362, and 0.0699, respectively.

### Comparison of SSTs

SSTs from moored buoys were used to evaluate three different water surface temperature algorithms (Fig. 1). Buoys J1 and J2 have been operated by the Jeonnam Sea Information Center (JSIC) since July 29, 2019. The locations of J1 and J2 are 126.41449° E, 35.0830° N, and 126.1977716° E, 35.111225° N, respectively (https://jnsi.jeonnam.go.kr/). Data are accessible to parties authorized by the JSIC. They provided hourly SST data observed by buoys. Buoy SST data obtained within the period that were closest to those of Landsat 8 were selected for comparison.

Buoy K1 is operated by the Korea Hydrographic and Oceanographic Agency (KHOA). The data can be downloaded from KHOA’s real-time ocean observation information system (http://www.khoa.go.kr). Buoy K1, located at 126.194255° E, 35.652458° N, provides a 30-min interval SST from January 1, 2015 to December 31, 2019.

Buoy K2, located at 125.8139° E, 35.6586° N, is operated by the Korea Meteorological Administration (KMA). Data were downloaded from the KMA Weather Data Service (https://data.kma.go.kr/). Buoy K2 provided hourly SST data from December 22, 2015. In this study, buoy data from December 22, 2015 to March 27, 2021 were compared with the SST data obtained from Landsat 8. SST values exceeding twice the standard deviation in each month for buoys K1 and K2 were removed.

The SST derived from Landsat 8 using three algorithms were compared with in-situ SST data at buoys. The root mean square error (RMSE) for the three SST and in-situ SST data were calculated for each buoy (Fig. 1). The three retrieval temperatures were in good agreement with the in-situ temperature. The RMSE was seasonally different (Supplementary Fig. S1). The maximum peak in June appeared in all the methods. The high RMSE in June might be affected by high relative humidity during the rainy season. The RMSE of ${\text{SST}}_{\text{R}}$ considering the humidity effect was lowest in July, whereas that of SST_J_ was lowest in March, April, October, November, and December. The overall performance of ${\text{SST}}_{\text{J}}$ was optimal, except for the high humidity period, because ${\text{SST}}_{\text{J}}$ uses optimized coefficients through fitting between brightness temperatures and in-situ temperatures on the Korean coast, which includes our study area, whereas SST_R_ and ${\text{SST}}_{\text{V}}$ adopt common coefficients depending on water vapor regardless of region. This suggests that SST_J_ represents the open sea temperature and coastal water temperature. SST_J_ corresponds to the in-situ SST at all buoys. R^2^ was 0.99 at buoys J1 and J2, and 0.98 at buoys K1 and K2. This result suggests that SST_J_ represents open sea temperature and coastal water temperature.

### Heat exchange between tidal flat and sea water

We can assume that the ratio between the horizontal SST gradients and the depth change is the same along both lines, where the effects of the river and current are not significant. The relationship between the horizontal water temperature gradient and the horizontal depth gradient can be expressed as follows:

5 $$\frac{{\text{W}}_{\text{grad}1}}{{d}_{grad1}}=\frac{{\text{W}}_{\text{grad}2}}{{d}_{grad2}},$$

where, ${\text{w}}_{\text{grad}1}$ and ${\text{w}}_{\text{grad}2}$ are the horizontal water temperature gradients (°C/m) along lines L1 and L2, respectively, and ${d}_{\text{grad}1}$ and ${d}_{\text{grad}2}$ are the horizontal depth gradients(m/m) along lines L1 and L2, respectively.

The additional heat flux per unit area (m^2^) for line L1 relative to line L2 was calculated using Eq. (6).

6 $${\Delta \text{q}}={\Delta {\text{T}}}\times \text{C}\times \text{m},$$

where, $\Delta$ q (J/m^2^) is the required heat sink or source, m (g) is the mass of seawater, and $\Delta$ T (°C) is the relative water temperature difference between the two onshore endpoints in both lines. The mass value per unit area at a depth of 4.9 m at the red point of L1 in Fig. 4a was used. C is the specific heat of water and is 4.184 J/g °C. $\Delta$ T was calculated as follows:

7 $$\Delta T= \left({\text{w}}_{\text{grad}1}-\left(\frac{{w}_{grad2}}{{d}_{grad2}}\times {d}_{grad1} \right)\right)\times lengt{h}_{L1}.$$

$lengt{h}_{L1}$ is the length of line L1. The temperature dependence of the water depth along both lines was assumed to be the same as that in previous studies^5,22,24^.

## Supplementary Information

Supplementary Figures.

## Data Availability

The data availability is outlined in “Methods” section. Correspondence and requests for materials should be addressed to S.-T.L and Y.-K.C.
