# Supplementary material for: Immense variability in the sea surface temperature near macro tidal flat revealed by high-resolution satellite data (Landsat 8)
Source: Sci Rep. 2022 Jan 7;12:248. doi: 10.1038/s41598-021-04465-4 (PMC8741900; doi:10.1038/s41598-021-04465-4)
Supplement: Supplementary file 1 — Supplementary Figures. [file 41598_2021_4465_MOESM1_ESM.docx]

**Supplementary Figures.
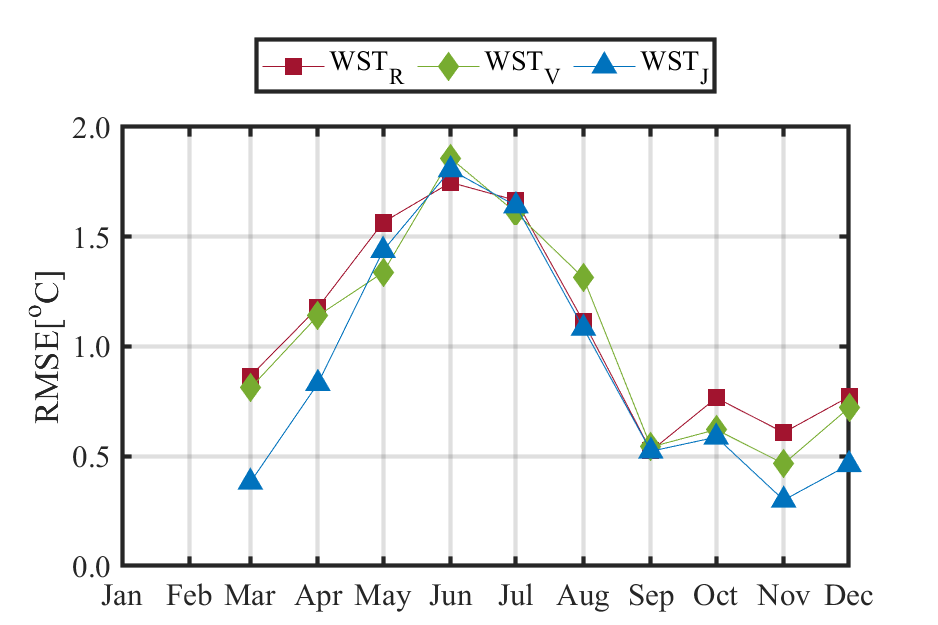
**

**Figure. S1.** RMSE between WST derived from Landsat 8 and SST observed at buoy K1 in each month from 2014 to 2021.


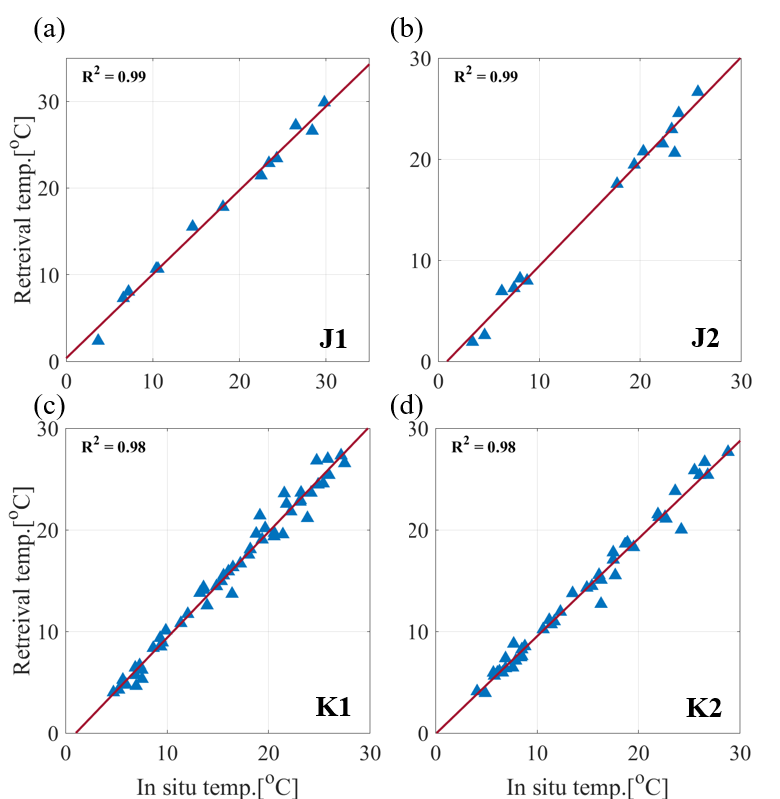


**Figure. S2.** Scatter plots of in-situ temperature and $\mathrm{WST}_{J}$ from Landsat 8 at buoys (a) J1, (b) J2, (c) K1, and (d) K2.


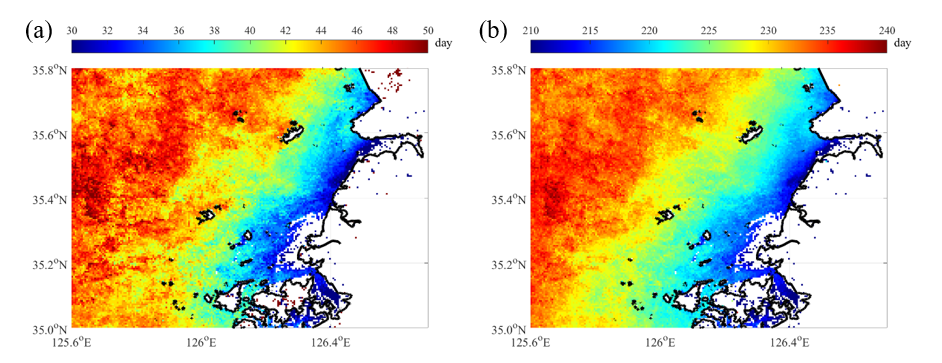


Figure. S3. Spatial distribution of annual (a) minimum temperature day and (b) maximum temperatures day. Figures were generated by S.-T.Lee using MATLAB R2020a (<http://www.mathworks.com>).
